# Palmitic Acid Reduces the Autophagic Flux and Insulin Sensitivity Through the Activation of the Free Fatty Acid Receptor 1 (FFAR1) in the Hypothalamic Neuronal Cell Line N43/5

**DOI:** 10.3389/fendo.2019.00176

**Published:** 2019-03-26

**Authors:** María Paz Hernández-Cáceres, Lilian Toledo-Valenzuela, Francisco Díaz-Castro, Yenniffer Ávalos, Paulina Burgos, Carla Narro, Daniel Peña-Oyarzun, Jasson Espinoza-Caicedo, Flavia Cifuentes-Araneda, Fernanda Navarro-Aguad, Cecilia Riquelme, Rodrigo Troncoso, Alfredo Criollo, Eugenia Morselli

**Affiliations:** ^1^Laboratory of Autophagy and Metabolism, Department of Physiology, Faculty of Biological Sciences, Pontificia Universidad Católica de Chile, Santiago, Chile; ^2^Autophagy Research Center, Santiago, Chile; ^3^Research Laboratory of Nutrition and Physical Activity, Institute of Nutrition and Food Technology, Universidad de Chile, Santiago, Chile; ^4^Advanced Center for Chronic Diseases and Center for Molecular Studies of the Cell, Universidad de Chile, Santiago, Chile; ^5^Facultad de Odontología, Instituto de Investigación en Ciencias Odontológicas, Universidad de Chile, Santiago, Chile; ^6^Laboratory of Differentiation and Pathology, Department of Cell and Molecular Biology, Faculty of Biological Sciences, Pontificia Universidad Católica de Chile, Santiago, Chile

**Keywords:** saturated fatty acids, G-protein coupled receptor 40, central nervous system, AKT, insulin resistance, autophagy, glucose uptake

## Abstract

Chronic consumption of high fat diets (HFDs), rich in saturated fatty acids (SatFAs) like palmitic acid (PA), is associated with the development of obesity and obesity-related metabolic diseases such as type II diabetes mellitus (T2DM). Previous studies indicate that PA accumulates in the hypothalamus following consumption of HFDs; in addition, HFDs consumption inhibits autophagy and reduces insulin sensitivity. Whether malfunction of autophagy specifically in hypothalamic neurons decreases insulin sensitivity remains unknown. PA does activate the Free Fatty Acid Receptor 1 (FFAR1), also known as G protein-coupled receptor 40 (GPR40); however, whether FFAR1 mediates the effects of PA on hypothalamic autophagy and insulin sensitivity has not been shown. Here, we demonstrate that exposure to PA inhibits the autophagic flux and reduces insulin sensitivity in a cellular model of hypothalamic neurons (N43/5 cells). Furthermore, we show that inhibition of autophagy and the autophagic flux reduces insulin sensitivity in hypothalamic neuronal cells. Interestingly, the inhibition of the autophagic flux, and the reduction in insulin sensitivity are prevented by pharmacological inhibition of FFAR1. Our findings show that dysregulation of autophagy reduces insulin sensitivity in hypothalamic neuronal cells. In addition, our data suggest FFAR1 mediates the ability of PA to inhibit autophagic flux and reduce insulin sensitivity in hypothalamic neuronal cells. These results reveal a novel cellular mechanism linking PA-rich diets to decreased insulin sensitivity in the hypothalamus and suggest that hypothalamic autophagy might represent a target for future T2DM therapies.

## Introduction

Obesity is currently considered a global epidemic both in developed and developing countries. Excessive accumulation of body fat promotes obesity-associated metabolic dysfunctions, such as insulin resistance, type II diabetes mellitus (T2DM), cardiovascular diseases, neurodegeneration, and certain cancers (1).

Consumption of high fat diets (HFDs), and especially of diets high in saturated fatty acids (SatFAs) such as the palmitic acid (PA), increases body weight and is associated with the onset of several metabolic diseases, such as insulin resistance and T2DM (2, 3). Previous studies from our and other groups show that SatFAs accumulate in the central nervous system (CNS), specifically in the hypothalamus (4–7). Interestingly, fatty acid (FA) sensing neurons reside in the hypothalamus and play important roles in feeding behavior, as well as lipid and glucose metabolism. Due to their critical role in the regulation of energy homeostasis, these neuronal populations have been implicated in development of obesity and T2DM (8, 9).

Macroautophagy, hereafter referred to as “autophagy,” is a conserved adaptive mechanism that maintains the balance between synthesis, degradation, and recycling of cellular components (10). Autophagy supports cellular homeostasis and studies in multiple cell types have demonstrated autophagic dysregulation following chronic consumption of HFDs (11). Previous studies from our and other groups have shown that hypothalamic autophagic flux is defective in rodents chronically fed with HFDs (12, 13). Consistently, previous work demonstrates that the inhibition of autophagy in proopiomelanocortin (POMC) neurons leads to metabolic disorders, including obesity and insulin resistance (14–16); however the mechanism by which this occurs is still largely unknown.

Evidence exists of crosstalk between autophagy and insulin signaling pathways. For example, hyperinsulinemia reduces the autophagic response in different peripheral tissues and cell types (17–20), whereas inhibition of autophagy (by autophagy related gene 7 -ATG7- tissue-specific knockout) promotes insulin resistance in liver tissue (21) and reduces insulin secretion in pancreatic β-cells (22, 23). However, whether inhibition of autophagy directly reduces neuronal insulin sensitivity is currently unknown.

The Free Fatty Acid Receptor 1 (FFAR1), also known as G protein-coupled receptor 40 (GPR40), is a seven-transmembrane domain receptor activated by medium and long-chain fatty acids, including PA (24, 25). Previous studies in GPR40^−/−^ mice show that this receptor plays a significant role in the chain of events linking obesity and metabolic disorders, as GPR40^−/−^ mice are protected from obesity-induced hyperinsulinemia, hyperglycemia, and glucose intolerance (26). Moreover, FFAR1 is required to mediate the insulin response to FA in the pancreas (27–29). However, its function in the hypothalamus, specifically in the context of autophagy and insulin sensitivity, is not known.

Here, we demonstrate that exposure to the SatFA PA inhibits the autophagic flux and reduces insulin sensitivity in N43/5 cells, a hypothalamic neuronal cell line. Furthermore, our findings show that autophagy malfunction promotes insulin resistance in hypothalamic neuronal cells. In addition, our data suggest FFAR1 may link PA with reduced insulin sensitivity and impaired autophagic flux in hypothalamic neuronal cells. Altogether, these results suggest activation of autophagy should be considered as treatment for insulin resistance.

## Materials and Methods

### Cell Culture and Treatments

N43/5 cells (Cellutions Biosystems) were cultured in Dulbecco's modified eagle medium (DMEM) high glucose (11995-040, Gibco, USA) supplemented with 10% of fetal bovine serum (FBS) (10437028, Gibco), 100 U/ml penicillin streptomycin (15140122, Gibco) and maintained at 37 °C with 5% CO2. To evaluate the changes in the autophagic flux in response to PA exposure, cells were incubated with DMEM high glucose supplemented with 2% of FBS 24 h before treatments and then exposed to 100 μM PA (P0500, Sigma-Aldrich, St. Louis, MO, USA) conjugated to fatty acid-free bovine serum albumin (BSA) (152401, MP Biomedicals, Santa Ana, CA, USA). BSA treatment was used as control. To assess the autophagic flux and insulin response, cells were incubated at the time points indicated in each experiment with the autophagic flux inhibitor Bafilomycin A1 (BafA1, 100 nM) (B1793, Sigma-Aldrich) or with its vehicle DMSO (BM-0660, Winkler). To determine the effect of 6-h treatment with PA or BafA1 on insulin signaling, cells were serum starved in medium DMEM/F-12 (11330-32, Gibco) overnight prior to treatments. Then, cells were co-treated with insulin (1 nM) (I0516, Sigma-Aldrich) or phosphate-buffered saline (PBS). To evaluate the involvement of FFAR1 in PA-mediated inhibition of the autophagic flux or reduction in insulin signaling, cells were pre-incubated with the FFAR1 antagonist GW1100 (1 μM; CAS 306974-70-9, Calbiochem, San Diego, CA, USA) for 20 min or with its vehicle (DMSO), followed by co-incubation with PA or BSA at the indicated time point, depending on the readouts.

### Animals

Animal care and procedures were approved by the Ethical Committee of the Pontificia Universidad Católica de Chile. Male C57BL/6 mice were housed in a temperature-controlled environment in groups of two to four at 22–24°C using a 12 h light/dark cycle. Mice were fed a standard chow (Prolab® RMH3000).

### Animals Gavage

Mice (n = 3–5/group) received an intragastric gavage of nutritionally complete diet as described by Benoit et al. (30). Briefly, the PA diet contains 20 g fat/100 g diet (19 g of ethyl palmitate dissolved in medium-chain triglyceride and 1 g of soybean oil to provide essential fatty acids), while the control diet contains 3 g ethyl palmitate and 1 g soybean oil/100 g diet. This liquid diet was injected into the stomach of the mice using a gavage-feeding needle. Mice were given 3 equally sized feedings daily at 7:00 AM, 12:00 PM, and 5:00 PM for 3 days. At the end of day 3, mice were sacrificed 3 h after the final gavage. Brains were removed, hypothalami dissected and stored in RNAlater (Ambion, Life Technologies) at 4 °C. 24 h later, tissues were homogenized in 1 ml of TRIzol (Ambion, Life Technologies) and RNA was extracted using the RNeasy Kit (Qiagen Sciences, Inc., Germantown, MD, USA) according to the manufacturer's instructions. Total RNA (1 μg) was reverse transcribed using the SuperScript III First-Strand Synthesis System (Invitrogen) according to the manufacturer's instructions and cDNA was used for RT-PCR assays.

### siRNA Transfections

Cells were cultured in six-well plates and transfected at 50% confluence with siRNAs targeting murine Beclin 1 (BECN1) (SASI_Mm01_00048143, Sigma-Aldrich) or murine autophagy related gene 7 (ATG7) (SASI_Mm01_00044616, Sigma-Aldrich). Transfection was performed using Lipofectamine RNAiMAX® Transfection Reagent (Invitrogen, Carlsbad, CA, USA) according to the manufacturer's instructions. As negative control, cells were incubated with Lipofectamine RNAiMAX® Transfection Reagent only. 48 h after siRNA transfection, cells were treated as indicated or directly lysed for protein or RNA extraction.

### Western Blot Analysis

Cells were lysed in RIPA buffer and 30–40 μg of denatured proteins from each sample were resolved in 8–12% SDS-PAGE. Gels were transferred to nitrocellulose membranes and incubated with 5% BSA (BM-0150, Winkler, RM, Chile)-tris-buffered saline-0.1% Tween-20 (TBS-T) to block nonspecific binding. Membranes were incubated with the primary antibodies anti LC3A/B (4108, Cell Signaling Technology, Danvers, MA, USA), SQSTM1 (H00008878-M01, Abnova, Jhouzih St., Taipei, Taiwan), p-Insulin Receptor β (Tyr1150/1151) (3024, Cell Signaling Technology), IR (ab131238, Abcam), p-AKT (Ser473) (9271, Cell Signaling Technology), AKT (9272, Cell Signaling Technology), BECN1 (H-300; sc-11427, Santa Cruz Biotechnology, Inc., Dallas, Texas, USA), at dilution of 1:1,000 in 5% BSA-TBS-T overnight on a rocking platform at 4 °C. Then, membranes were washed 3 times for 10 min in TBS-T and revealed with the appropriate horseradish peroxidase-labeled secondary antibodies (Goat Anti-Mouse IgG (H + L)-HRP Conjugate, 1706516; Goat Anti-Rabbit IgG (H + L)-HRP Conjugate, 1706515; Bio-Rad, CA, USA) and the chemiluminescent substrate. GAPDH (1:1000; sc-365062, Santa Cruz Biotechnology, Inc.) and β-actin (1:10,000; A1978, Sigma-Aldrich) were used as loading control. To evaluate insulin signaling the same samples were run on parallel gels, one for p-AKT and the other for AKT. Analysis of data was performed by comparing p-AKT vs. β-actin and AKT vs. β-actin. The obtained ratios were analyzed.

### Immunofluorescence and Fluorescence Microscopy

For fluorescence microscopy determinations in N43/5 cells, cells cultured on coverslips were fixed with cold methanol (−20 °C) for 10 min. Cells were blocked in 3% BSA in PBS for 1 h and then incubated with the following primary antibodies overnight at 4 °C. The primary antibodies used are LC3A/B (1:250; Cell Signaling Technology), p62/SQSTM1 (1:300; Abnova). Primary antibodies staining was followed by conjugation with its respective secondary antibody (1:300; Alexa Fluor®, Life Technologies) for 1 h at room temperature. Nuclei were counterstained with Hoechst 33342 (10 mg/ml) (Molecular Probes, Eugene, OR, USA) or with ProLong® Gold Antifade Mountant (P36931, Molecular Probes, Eugene, OR, USA). Images were taken in an inverted fluorescence microscopy (Nikon Eclipse Ti, Tokio, Japon).

For fluorescence microscopy determinations on brain sections, mice were anesthetized and perfused with 10% formalin. Brains were dissected and post-fixed in 10% formalin for 24 h followed by treatment with 30% sucrose in PBS. Brain sections were cut at 30 μm using a Thermo Scientific HM 450 sliding microtome (Thermo Scientific). The sections were permeabilized in 0.01% Triton and blocked in 3% bovine BSA (BM-0150 Winkler) for 1 h. Brain sections were incubated overnight at 4 °C with the primary antibody against GPR40 (Y17; sc-28416, Santa Cruz Biotechnology, Inc.) in combination with either a marker of neurons (NeuN; MAB377, Millipore, Burlington, MA, USA), astrocytes (GFAP; G9269, Sigma-Aldrich) or microglia (IBA-1; 019-19741, Wako, USA), followed by conjugation with the respective secondary antibodies (Alexa Fluor, Life Technologies) for 1 h at room temperature. Sections were placed on gelatinized slides, mounted with VECTASHIELD anti-fading medium with DAPI (Vector Laboratories), and coverslipped. Pictures containing the arcuate nucleus (ARC) region of the hypothalamus were taken in a confocal fluorescence microscopy (Carl Zeiss, LSM700, Oberkochen, Germany).

### Real Time PCR (RT-PCR)

For analysis of gene expression, mice were anesthetized and decapitated. Tissues were stored in RNA*later* (Ambion, Life Technologies) at 4° C, and 24 h later, hypothalami dissected and then homogenized in 1 ml of TRIzol (Ambion, Life Technologies). RNA was extracted using the RNeasy Kit (Qiagen Sciences, Inc., Germantown, MD, USA) according to the manufacturer's instructions. Total RNA (1 μg) was reverse transcribed using the SuperScript III First-Strand Synthesis System (Invitrogen) according to the manufacturer's instructions.

For analysis of *Atg7* gene expression in N43/5 cells, RNA was extracted using the E.Z.N.A.® Total RNA Kit (OMEGA Bio-Tek, Norcross, GA, USA) according to the manufacture's indications. cDNA was synthesized using iScriptTM cDNA kit (Bio-Rad) from 1 μg of total RNA.

Quantitative PCR reactions were carried out on a Step One System Real Time PCR (Applied Biosystems) using Fast SYBR Green Master Mix (4385370, Life Technologies). Specific forward and reverse primers sequences used to evaluate gene expression are indicated in Supplemental Table 1. *Hprt1* was used as housekeeping gene. The ΔΔC_T_ method was used for relative quantification analysis.

### Intracellular Calcium Measurement

Intracellular calcium (Ca^2+^) was determined using a spectrofluorometric technique described by Darling R.A. and collaborators (31). N43/5 cells were loaded with 1.5 μM Fura 2-AM (Molecular Probes) for 45 min at 37 °C. Cells were excited at a wavelength of 340 and 380 nm in a Nikon Diaphot microscope equipped with a Photon Technology International spectrofluorometer (Lawrenceville, NJ). To confirm the presence of a functional FFAR1 in our cellular model, cells were pre-incubated with its antagonist GW1100 (1 μM) for 20 min. After 10 min of stabilization, the intensity ratio was continuously recorded and cultures were co-stimulated for 210 seconds with 100 μM PA or BSA. Fluorescence emission intensity was detected with a photomultiplier and analyzed using a fluorescence analysis program (FELIX version 1.1).

### 2-NBDG Uptake

To assess the insulin-dependent glucose uptake, N43/5 cells were stimulated with insulin 1 nM for 30 min and incubated with the fluorescent analog of glucose (2-NBDG, 300 mM) for 15 min at 37 °C as previously described (32). To evaluate the involvement of FFAR1 in PA-mediated inhibition of insulin-dependent glucose uptake, cells were pre-incubated with the FFAR1 antagonist GW1100. Cells were transferred to an inverted Nikon Ti Eclipse microscope equipped with 40X oil objective [numerical aperture, N.A. 1.3]. A Xenon lamp was coupled to the monochromator device (Cairn Research Ltd, Faversham, UK). Digital images were acquired by means of a cooled CCD camera (Hamamatsu ORCA 03, Japan). Images were quantified by ImageJ software (NIH, Bethesda, MD).

### Results and Statistical Analysis

Results are shown as mean ± SEM from at least 3 independent experiments. Two groups were compared using two-tailed Student's *t* tests. For more than two groups, one- or two-way ANOVA was used, as appropriate, followed by *post hoc* adjustment. All analyses were performed with GraphPad software (San Diego, CA, USA). *P* value of < 0.05 was considered statistically significant.

## Results

### Palmitic Acid Inhibits the Autophagic Flux in N43/5 Cells

**Figure 1:**
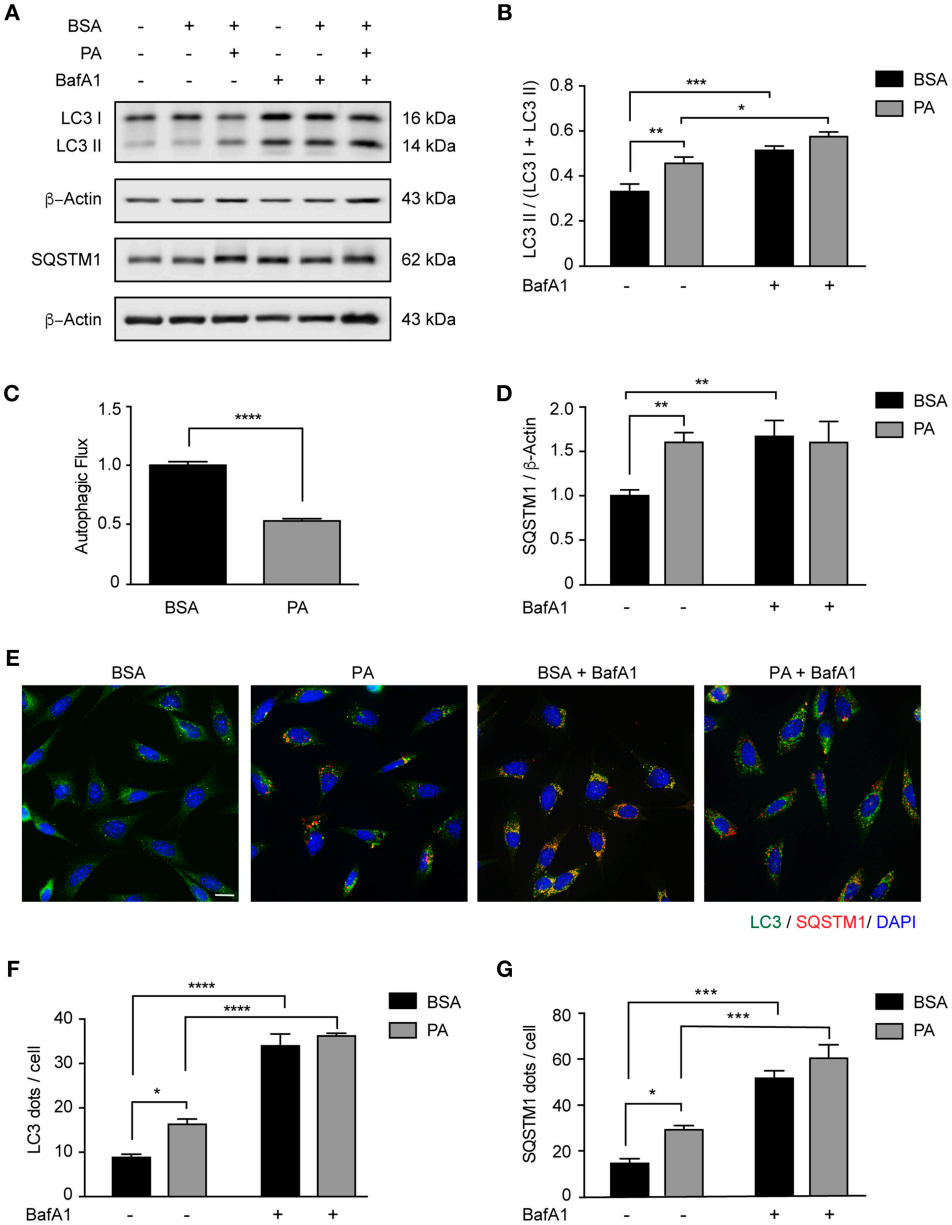
Palmitic acid inhibits the autophagic flux in N43/5 cells. **(A)** Representative western blot of LC3 and SQSTM1 levels in N43/5 cell lysates, incubated with vehicle (BSA) or PA (100 μM) for 6 h, in presence or absence of BafA1 (100 nM), with their respective quantifications **(B,D)**. **(C)** Autophagic flux calculated as the difference in LC3II levels in presence and absence of BafA1 (100 nM). **(E)** Representative images of N43/5 cells treated with BSA or PA (100 μM) for 6 h in presence or absence of BafA1 (100 nM) and stained against LC3 (green) and SQSTM1 (red). Nuclei are stained with DAPI (blue). Quantification of LC3 **(F)** and SQSTM1 **(G)** dots per cell. Size bar: 10 μm. Data are presented as mean ± SEM. Two ways ANOVA followed by Sidak's multiple comparisons test. ^*^p < 0.05, ^**^p < 0.01, ^***^p < 0.001, ^****^p < 0.0001. *n* = 3.

We and others have previously shown that autophagy is dysregulated *in vivo* in the hypothalamus of male mice chronically fed with HFDs (12, 13, 33). Interestingly, we also demonstrated that in the brain of mice fed with the same diet for 16 weeks, SatFAs are significantly increased (4, 6, 7). Among those PA represents the most abundant SatFA, thus, we decided to evaluate whether its increase affects basal autophagy in hypothalamic neurons. We exposed N43/5 cells, a model of POMC neurons (34), to 100 μM PA, a concentration of PA similar to the one identified in the brain of obese mice chronically exposed to HFDs (7). As indicated by western blot, the levels of the autophagy receptor Sequestosome 1/p62 (SQSTM1), which is degraded during the autophagic process, increased compared to control (BSA vs. PA: ^**^p < 0.01; Figures 1A,D), suggesting PA inhibited autophagic flux. This result was supported by measuring levels of microtubule-associated protein light chain 3 II (LC3II) in presence and absence of Bafilomycin A1 (BafA1), a classic autophagic flux inhibitor which prevents fusion of autophagosomes and lysosomes (Figures 1A–C). PA exposure increased the levels of LC3II; however, addition of BafA1 had not effect (BSA vs. PA: ^**^p < 0.01; BafA1 vs. BafA1 + PA: non-significant; Figures 1A–C). Consistent with this, immunofluorescence studies in N43/5 cells stained against LC3 showed that treatment with PA stimulated the formation of autophagic puncta *per se* already 1 h following PA exposure (Figures 1E,F and Supplemental Figures 1A,B). However, exposure to BafA1 did not further enhance the PA-triggered induction of LC3 puncta (Figures 1E,F and Supplemental Figures 1A,B). Finally, in addition to LC3 puncta, PA treatment also increased the number of SQSTM1 puncta, already 1 h following PA treatment (Figures 1E,G and Supplemental Figures 1A,C). Importantly, the levels of *Atg5, Atg7, Atg16, Beclin 1, Fip200, and Gabarap* are not increased by PA exposure, and *Lc3* and *Sqstm1* levels, even if affected, are significantly increased only 6 and 4 h, respectively, following PA exposure (Supplemental Figures 1D–K), thus suggesting PA treatment specifically affects the autophagic flux.

Importantly, as PA is not the only SatFA increased in the brain of male mice chronically fed with HFDs, to determine if the inhibition of the autophagic flux is specific to PA or not, we evaluated the effect of stearic acid (SA) on autophagy on N43/5 cells. The results we obtained show also SA inhibits the autophagic flux (Supplemental Figure 2). Indeed, as indicated by western blot, the levels of SQSTM1 are significantly increased when compared to BSA (BSA vs. SA: ^*^p < 0.05; Supplemental Figures 2C,D). Consistent with this, the levels of LC3II, which are increased by SA *per se*, were not affected by BafA1 (BSA vs. SA: ^**^p < 0.01, BafA1 + BSA vs. BafA1 + SA: non-significant; Supplemental Figures 2A,B). Accordingly, SA stimulated the formation of autophagic puncta, as assessed by immunofluorescence (Supplemental Figures 2E,F) and exposure to BafA1 did not further enhance the SA-triggered induction of LC3 puncta (Supplemental Figures 2E,F). Finally, in addition to LC3 puncta, SA treatment also increased the number of SQSTM1 puncta (Supplemental Figures 2E,G). Altogether, these data indicate that SatFAs inhibit the basal rate of autophagy in N43/5 hypothalamic neurons.

### Palmitic Acid Reduces Insulin Sensitivity in N43/5 Cells

**Figure 2:**
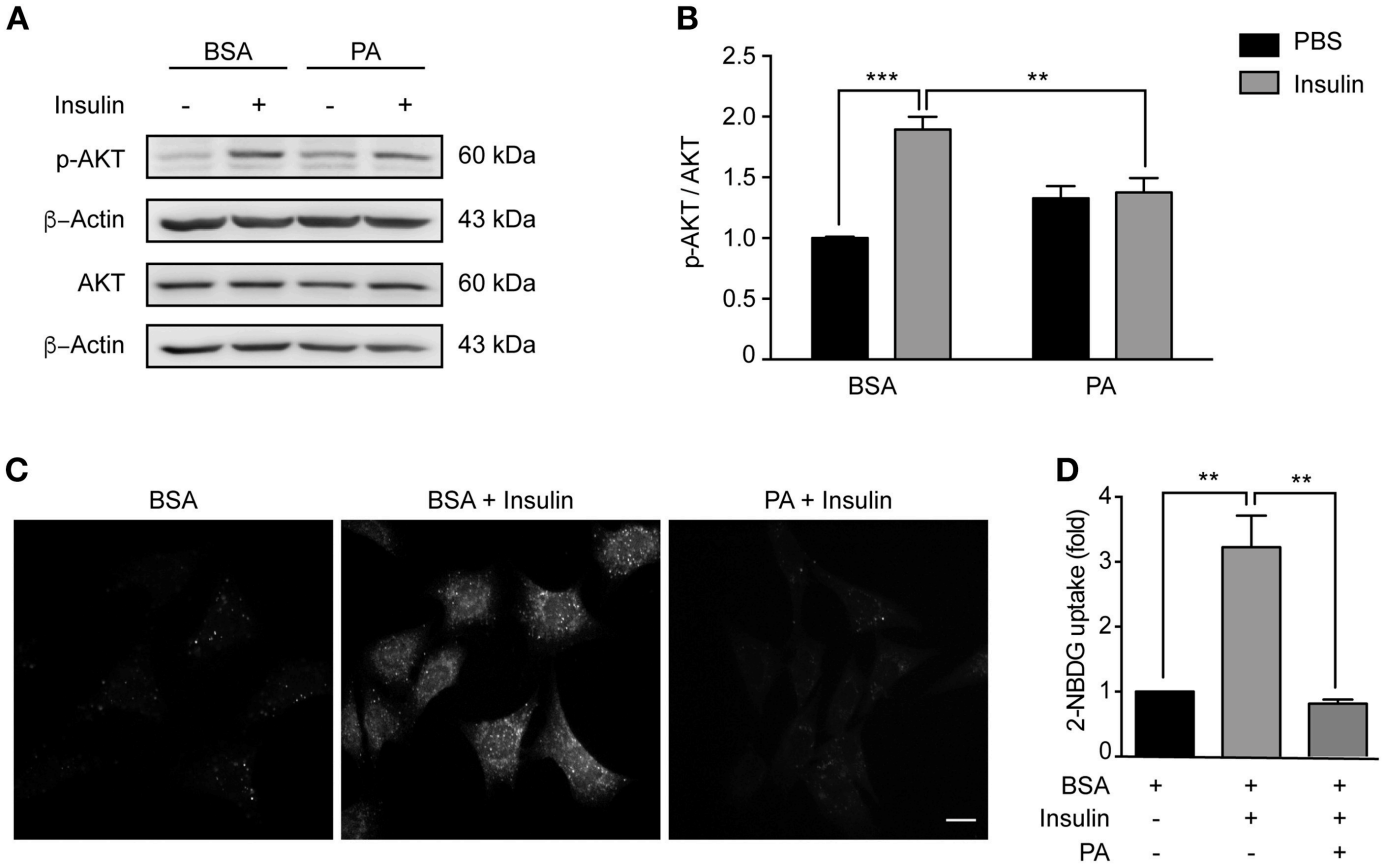
Palmitic acid reduces insulin sensitivity in N43/5 cells. **(A)** Representative western blot showing relative levels of AKT phosphorylation (Ser473) induced by 15 min of insulin or PBS treatment in N43/5 cells pre-incubated with PA (100 μM) or BSA for 6 h, with its respective quantification **(B)**. **(B)** Data are presented as mean ± SEM. Two ways ANOVA followed by Sidak's multiple comparisons test. ^**^p < 0.01, ^***^p < 0.001. *n* = 3. **(C)** Representative images of 2-NBDG uptake in N43/5 cells incubated with vehicle (BSA) or PA for 6 h and then stimulated with insulin 1 nM for 30 min, with its representative quantification **(D)**. Size bar: 10 μm. **(D)** Data are presented as mean ± SEM. One-way ANOVA followed by Holm-Sidak's multiple comparisons test. ^**^p < 0.01. *n* = 3.

Consumption of HFDs causes hypothalamic insulin resistance in rodents (35). Importantly, insulin activates neurons located in the arcuate nucleus of the hypothalamus (such as POMC neurons), thereby reducing food intake. In conditions of chronic caloric excess (as occurs in HFDs feeding), insulin resistance develops and contributes to pathological weight gain (36). Moreover, consumption of HFDs increases PA levels in the hypothalamus (4, 5), and intracerebroventricular injection of PA blunts hypothalamic insulin signaling (30). Here, we evaluated the sensitivity of our cellular model to insulin, and whether PA exposure could reduce insulin sensitivity in the same cell type. Following exposure to insulin, levels of AKT phosphorylation (p-AKT) increased (Figures 2A,B), showing that N43/5 hypothalamic neuronal cells are, indeed, sensitive to insulin. Critically, PA treatment reduced phosphorylation levels (BSA + Ins vs. PA + Ins: ^**^p < 0.01; Figures 2A,B) and consistently, PA exposure inhibited insulin-induced glucose uptake (BSA + Ins vs. PA + Ins: ^**^p < 0.01; Figures 2C,D). Importantly, these effects seem not be specific of PA, as also SA exposure reduces insulin sensitivity, as indicated by p-AKT levels in presence and absence of insulin (BSA + Ins vs. SA + Ins: ^****^p < 0.0001; Supplemental Figure 3). Altogether these results indicate SatFAs reduce insulin sensitivity in N43/5 cells.

### Inhibition of Autophagy Reduces Insulin Sensitivity in N43/5 Cells

**Figure 3:**
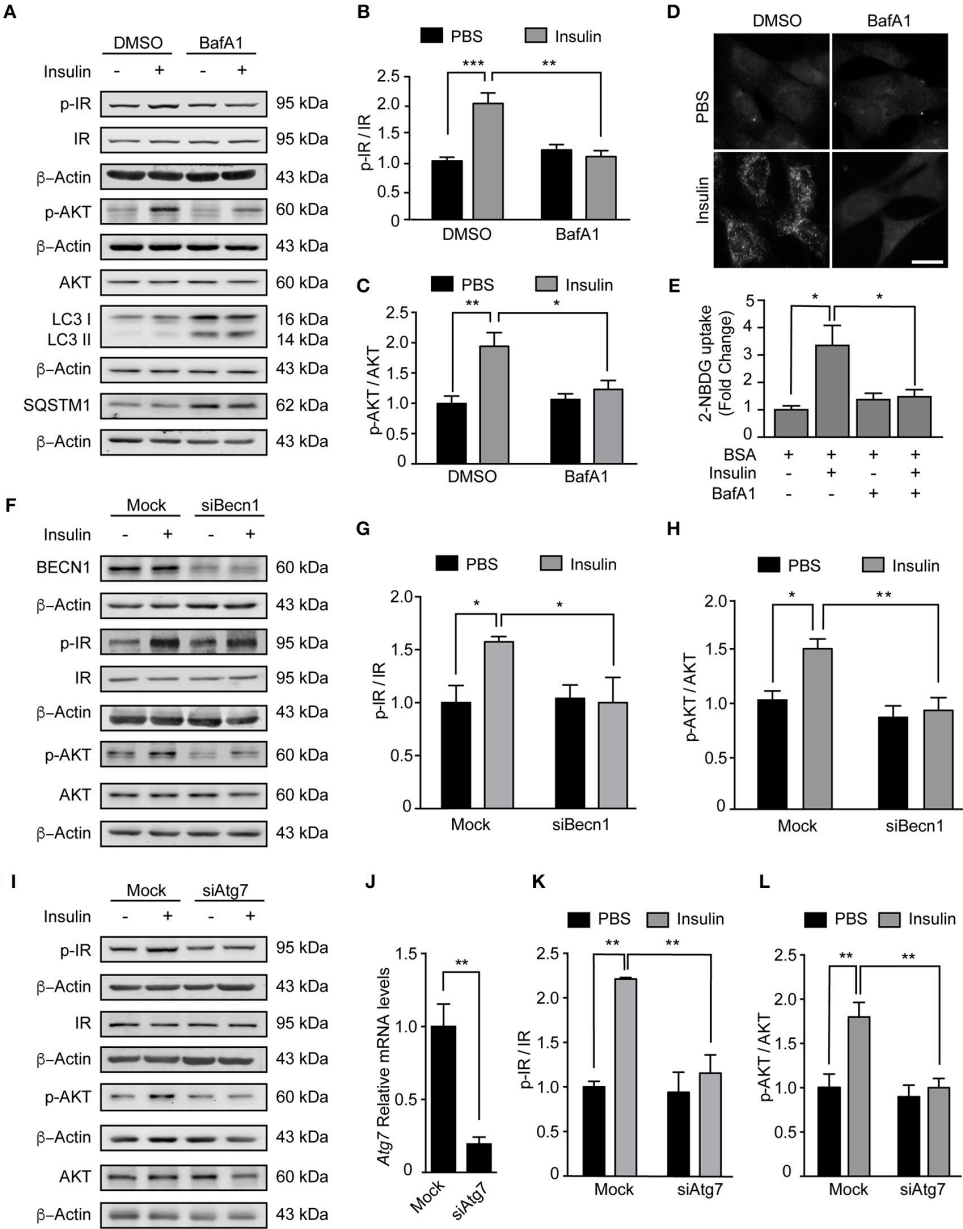
Inhibition of autophagy reduces insulin sensitivity in N43/5 cells. **(A)** Representative western blots of the indicated proteins of N43/5 cells pre-incubated with BafA1 (100 nM) or its vehicle (DMSO) during 6 h and then stimulated with insulin for 3 min to evaluate IR phosphorylation (Tyr1150/1115) or 15 min to evaluate AKT phosphorylation. **(B,C)** Quantification of IR (Tyr1150/115) and AKT (Ser473) phosphorylation in cells exposed to the treatments as indicated in **(A)**. **(B,C)** Data are presented as mean ± SEM. Two ways ANOVA followed by Sidak's multiple comparisons test. ^*^p < 0.05, ^**^p < 0.01, ^***^p < 0.001. *n* = 3. **(D)** Representative images and quantification **(E)** of 2-NBDG uptake in N43/5 cells pre-incubated with BafA1 (100 nM) or its vehicle (DMSO) during 6 h and then stimulated with insulin for 30 min. Size bar: 10 μm. **(E)** Data are presented as mean ± SEM. One-way ANOVA followed by Holm-Sidak's multiple comparisons test. ^*^p < 0.05. *n* = 3. Representative blot of the indicated proteins of N43/5 cells transfected with siRNA against BECN1 **(F)** or ATG7 **(I)** followed by insulin or PBS treatment for 3 min to evaluate IR phosphorylation or 15 min to evaluate AKT phosphorylation, with its respective quantifications **(G,H,K,L)**. **(G,H,K,L)** Data are presented as mean ± SEM. Two ways ANOVA followed by Sidak's multiple comparisons test. ^*^p < 0.05, ^**^p < 0.01. *n* = 3. **(J)** mRNA levels of *Atg7* in N43/5 cells transfected with a siRNA to downregulate ATG7. As control condition, cells were incubated with Lipofectamine RNAiMAX reagent only (Mock). Data are presented as mean ± SEM. Unpaired *t* test. ^**^p < 0.01. *n* = 3.

Our data indicate PA inhibits the autophagic flux and reduces insulin sensitivity. In the next series of experiments, we determined if this response is specific to PA or if other compounds that inhibit the autophagic flux also affect the insulin response. First, we exposed N43/5 cells to BafA1 or vehicle for 6 h and added insulin during the last 3 or 15 min. While insulin treatment increased p-Insulin Receptor (IR) and p-AKT levels (3 and 15 min following insulin treatment, respectively), again suggesting N43/5 cells are sensitive to insulin, pretreatment with BafA1 prevented the increase in their phosphorylation (Ins vs. BafA1 + Ins: ^**^p < 0.01 for p-IR and ^*^p < 0.05 for p-AKT; Figures 3A–C). Importantly, the reduction in insulin sensitivity is confirmed by the reduction in glucose uptake, which is blunted by BafA1 treatment (Ins vs. BafA1 + Ins: ^*^p < 0.05; Figures 3D,E). No differences were seen in *Glut4* expression, suggesting the effect of BafA1 does not occur at the transcriptional level (Supplemental Figure 4). In addition, BafA1 exposure increased the levels of LC3II and SQSTM1 (Figure 3A), suggesting that inhibition of the autophagic flux reduces the insulin response in hypothalamic neuronal cells.

Then, we assessed if inhibition of autophagy, and not only the inhibition of the autophagic flux, could affect the response to insulin in our cellular model. To do this, we downregulated the expression of two autophagy essential genes *Beclin1* and *Atg7* (Figures 3F,J) and evaluated the levels of IR and p-AKT in response to insulin treatment. Interestingly, downregulation of both autophagy essential genes significantly reduced the levels of p-IR and p-AKT following insulin treatment (Ins vs. siBeclin + Ins: ^*^p < 0.05 for p-IR and ^**^p < 0.01 for p-AKT. Ins vs. siAtg7 + Ins: ^**^p < 0.01 for p-IR and p-AKT; Figures 3F–L). *Glut4* expression levels were increased following *Atg7* downregulation, while no differences were identified following BafA1 exposure or *Beclin1* downregulation (Supplemental Figure 4). Altogether these results indicate inhibition of autophagy, by downregulation of autophagy essential genes, reduces insulin sensitivity of N43/5 cells *in vitro*.

### Palmitic Acid Activates FFAR1 in N43/5 Cells

**Figure 4:**
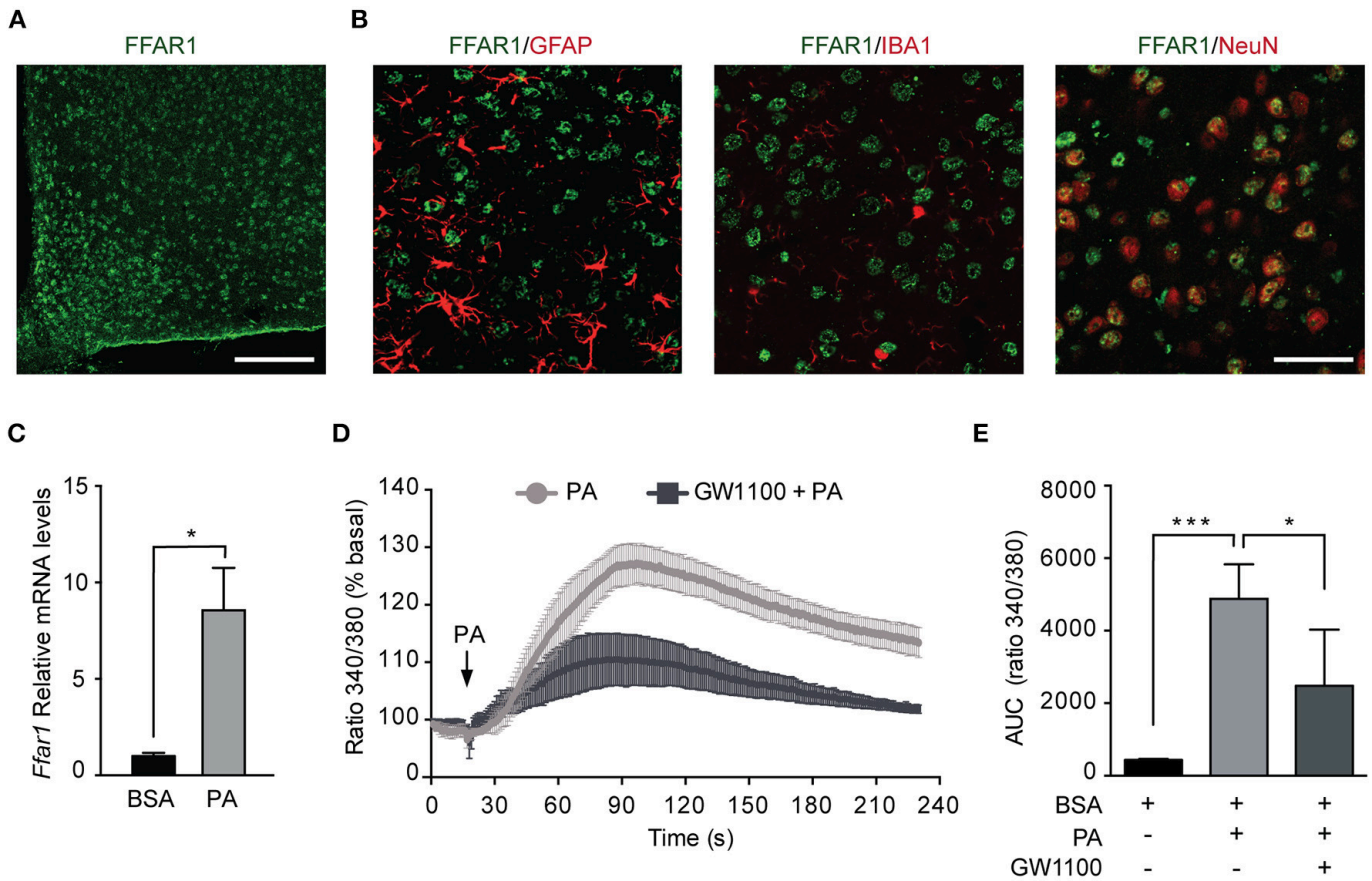
Palmitic acid activates FFAR1 in N43/5 cells. **(A)** Representative confocal images showing FFAR1 immunoreactivity in the arcuate (ARC) nucleus of the hypothalamus of male mice. Scale bar: 125 μM. **(B)** Representative confocal images showing colocalization between FFAR1 and glial fibrillary acidic protein (GFAP), which stains astrocytes, or ionized calcium-binding adapter molecule 1 (IBA-1), which stains microglia or neuronal nuclei (NeuN), which stains neurons, in the hypothalamus. Scale bar: 25 μM. **(C)** mRNA levels of *Ffar1* in hypothalamic tissue in mice gavaged with BSA or PA for 3 days. Data are presented as mean ± SEM. Unpaired *t* test. BSA, *n* = 3; PA, *n* = 4. **(D)** Representative plot showing the percent increase in intracellular Ca^2+^ levels induced by PA (100 μM), in the presence or absence of FFAR1 antagonist GW1100 (1 μM). **(E)** Area under the curves (AUC) represented in **(D)**. Data are shown as mean ± SEM. One-way ANOVA followed by Holm-Sidak's multiple comparisons test. ^*^p < 0.05, ^***^p < 0.001. *n* = 4.

Given the increased PA in the hypothalamus of HFDs animals (5), we addressed whether PA affects the levels of FFAR1. Consistent with previous work (37, 38), we identified FFAR1 in hypothalamic neurons but not astrocytes or microglia (Figures 4A,B). To determine the effect of PA on FFAR1 levels we gavaged male mice with PA. Importantly, this experiment was performed only in male mice as the increase in PA concentration in the brain, and more specifically in the hypothalamus, was identified only in male mice (4–7). Our data showed *Ffar1* mRNA levels increased following PA gavage (BSA vs. PA ^*^p < 0.05; Figure 4C). Furthermore, consistently with the results we obtained in N43/5 cells, *Sqstm1* levels increased, while *Atg* genes were not affected (Supplemental Figure 5).

The activation of FFAR1 by FAs triggers the phospholipase C (PLC)/ Inositol trisphosphate (IP_3_) signaling pathway leading to Ca^2+^ release from the endoplasmic reticulum (24, 39). Thus, to determine if PA exposure activates FFAR1 in our cell model, we assessed intracellular Ca^2+^ levels following PA treatment. Intracellular Ca^2+^ levels were significantly increased in N43/5 cells after exposure to PA relative to vehicle (BSA vs. PA ^***^p < 0.001; Figures 4D,E). Importantly, this increase was significantly reduced in cultures exposed to GW1100, a specific FFAR1 antagonist, prior to PA treatment (PA vs. GW1100 + PA ^*^p < 0.05; Figures 4D,E). These data suggest that PA, among other factors, activates FFAR1 in N43/5 cells.

### FFAR1 Pharmacological Inhibition Restores Autophagy and Insulin Sensitivity

**Figure 5:**
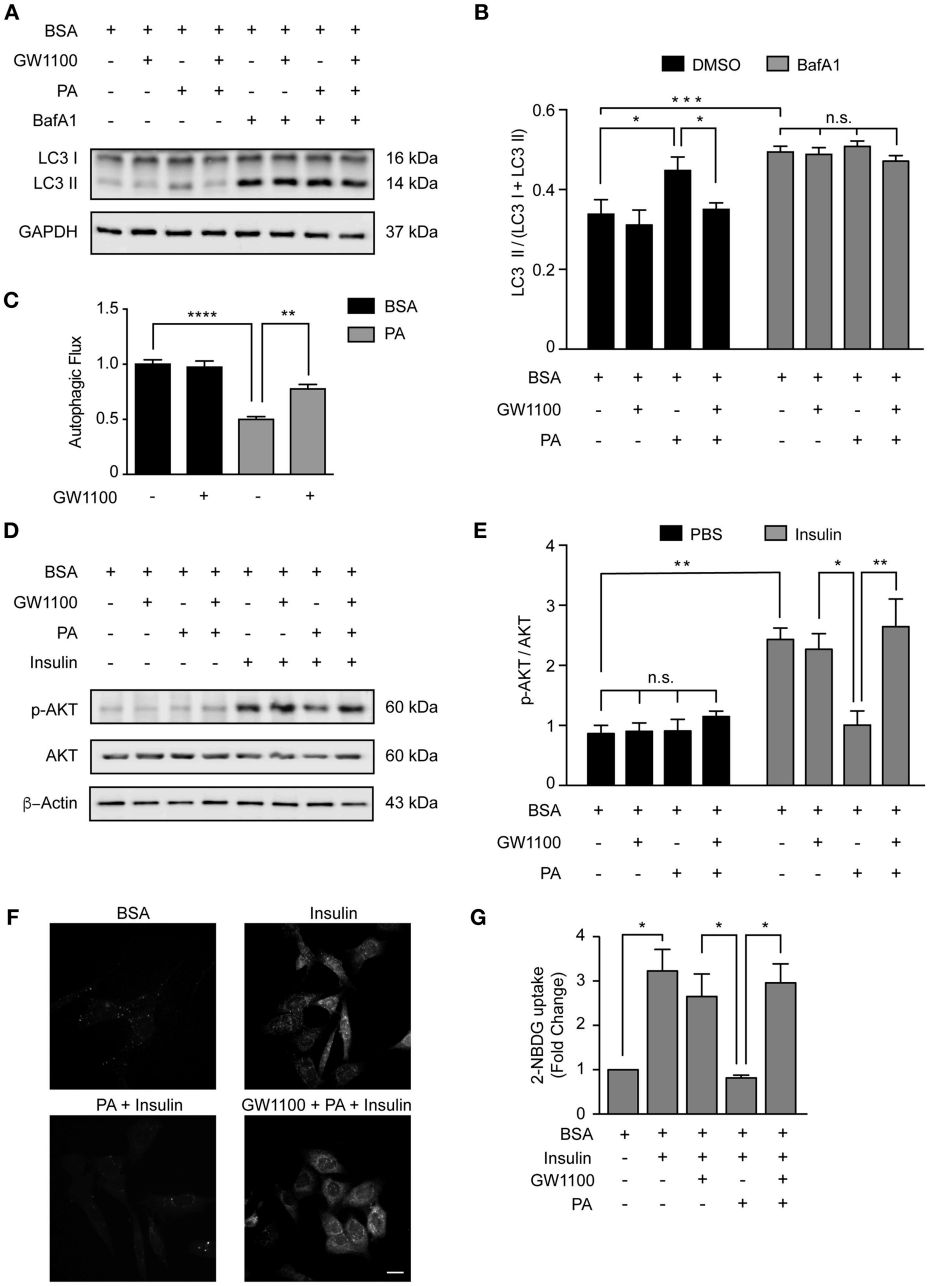
FFAR1 pharmacological inhibition restores autophagy and insulin sensitivity. **(A)** Representative western blot of LC3 levels in N43/5 cell lysates, incubated with vehicle (BSA), GW1100 (1 μM), or PA (100 μM) for 1 h as indicated, in presence or absence of BafA1 (100 nM), with its respective quantification **(B)**. **(B)** Data are presented as mean ± SEM. Two ways ANOVA followed by Sidak's multiple comparisons test. ^*^p < 0.05, ^***^p < 0.001. *n* = 3. In case of GW1100 and PA treatment, cells were pre-exposed to GW1100 for 20 min, followed by GW1100 and PA exposure for 1 h. **(C)** Autophagic flux calculated as the difference in LC3II levels in presence and absence of BafA1 (100 nM). Data are shown as mean ± SEM. One-way ANOVA followed by Holm-Sidak's multiple comparisons test. ^*^*p < 0.01, ^****^p < 0.0001. **(D)** Representative western blot of N43/5 cell lysates, incubated with vehicle (BSA), GW1100 (1 μM) or PA (100 μM) for 15 min in presence or absence of insulin (1 nM) for additional 15 min, as indicated, with its respective quantification **(E)**. In case of GW1100 and PA treatment, cells were pre-treated with GW1100 for 20 min, followed by GW1100 and PA exposure for 15 min. Data are presented as mean ± SEM. Two ways ANOVA followed by Sidak's multiple comparisons test. ^*^p < 0.05, ^**^p < 0.01. *n* = 3. **(F)** Representative images of 2-NBDG uptake in N43/5 cells incubated with vehicle (BSA), GW1100, PA and then stimulated with insulin 1 nM for 30 min, with its representative quantification **(G)**. Size bar: 10 μm. Data are shown as mean ± SEM. One-way ANOVA followed by Holm-Sidak's multiple comparisons test. ^*^p < 0.05. *n* = 3.

Our data indicate that PA inhibits the autophagic flux and activates FFAR1 (Figures 1, 4); thus, we determined if the activation of FFAR1 by PA is mechanistically involved in the inhibition of the autophagic flux. We exposed N43/5 cells to the FFAR1 antagonist GW1100 prior to PA treatment. GW1100 pretreatment reduced the levels of LC3II in cells pre-exposed to PA (PA vs. GW1100+PA ^*^p < 0.05; Figures 5A,B). As we previously showed that PA inhibits the autophagic flux (Figure 1), these results suggest FFAR1 inhibition restores the autophagic flux in cells treated with PA (Figure 5C).

Interestingly, pharmacological inhibition of FFAR1 activity in cells exposed to PA also restored insulin sensitivity in N43/5 cells. Indeed, PA exposure reduced p-AKT levels when cells were stimulated with insulin (Figures 2, 5D,E). However, p-AKT levels in cells treated with insulin were similar between control cells (treated with BSA) and cells pre-treated with GW1100, prior to PA exposure (PA + Ins vs. GW1100 + PA + Ins ^*^p < 0.05; Figures 5D,E). Consistently, glucose uptake, significantly reduced by PA treatment, is restored by GW1100 pretreatment (Figures 5F,G). Again, no significant differences were seen in *Glut4* mRNA levels following treatments (Supplemental Figure 6). These data indicate FFAR1 pharmacological inhibition promotes insulin sensitivity in N43/5 cells exposed to PA.

## Discussion

Here, we demonstrate that exposure to the SatFA PA inhibits the autophagic flux and reduces insulin sensitivity in N43/5 hypothalamic neurons (Figures 1, 2). Furthermore, we show that inhibition of autophagy and the autophagic flux reduces insulin sensitivity in the same cellular model (Figure 3). Lastly, our data indicate that PA activates FFAR1 in hypothalamic neuronal cells (Figure 4) and that the inhibition of the autophagic flux and the reduction in insulin sensitivity are prevented by pharmacological inhibition of FFAR1 (Figure 5).

Chronic consumption of HFDs promotes the accumulation of PA in the hypothalamus of male mice (4–7); thus, we decided to determine whether this increase in PA might affect autophagy, which also appears dysfunctional in the hypothalamus following chronic consumption of HFDs. Importantly, previous studies indicate autophagy malfunction in hypothalamic POMC neurons contributes to obesity-associated metabolic dysfunctions, including increased plasma insulin levels, hyperglycemia, glucose intolerance, impaired lipolysis, and leptin resistance (14–16). Taken together, these data suggest that HFDs-induced SatFAs accumulation in the CNS in male mice promotes a vicious cycle where autophagy inhibition might further enhance obesity-associated diseases. In this context, our data indicate that, at least in our cellular model, exposure to PA inhibits the autophagic flux, a condition that also affects insulin sensitivity. Future research, should confirm these data in *in vivo* models. Special attention should be paid to assess the possible sexual dimorphism is this response, especially considering previous studies indicating the sex-dependent function of POMC neurons in the regulation of energy homeostasis (40, 41).

The existence of a crosstalk between autophagy and insulin sensitivity has been previously suggested and identified in peripheral metabolic tissues (22, 23, 42). Interestingly, it was recently demonstrated that autophagy degrades insulin-containing vesicles in β-cells of autophagy-hyperactive mice, whereas in insulin-sensitive cells, autophagy enhances insulin response (42). Indeed, induction of autophagy, by different means, in skeletal muscle, hepatocytes, podocytes and adipocytes (43–45), stimulates insulin sensitivity; suggesting increased autophagy might be a general mechanism to boost insulin response. Despite this, there are no reports that determine if this crosstalk also occurs in the CNS, or specifically, in the hypothalamus where insulin sensitive neurons key in the regulation of food intake and peripheral glucose homeostasis reside. In the present study, we evaluated if inhibition of autophagy or autophagic flux blockade in a hypothalamic neuronal cell line affects insulin response. Our data indicate that this might be the case, as downregulation of different autophagy essential genes (*Atg7* and *Beclin1*), as well as inhibition of the autophagic flux using a classic autophagy inhibitor (Bafilomycin A1), reduced the ability of the neuron to respond to insulin, as indicated by IR and p-AKT level and the reduction in glucose uptake following Bafilomycin A1 exposure. How this might be occurring has not been elucidated; however, a possibility is that, by inhibiting autophagy we prevent the degradation of negative regulators of the insulin signaling pathway, such as the protein phosphatase and tensin homolog (PTEN), which can be degraded by the autophagic/lysosomal pathway (46). In addition, based on these results, it is tempting to speculate that the maintenance of the autophagic balance in hypothalamic neurons might be key in the regulation of peripheral glucose homeostasis, as well as food intake. Future studies in neuron-specific transgenic mouse models need to be performed to confirm this hypothesis *in vivo*.

Sensing lipids is a mechanism through which the hypothalamus controls energy balance. Brain lipid sensing is important to control feeding behavior, hepatic glucose production, and insulin secretion (8). Fatty acids sensitive neurons can increase/decrease their activity in response to FAs (8). Different types of proteins can act as receptors of fatty acids, such as fatty acid transporters, fatty acid binding proteins, and fatty acid receptors. Among those, we focused our attention on FFAR1, the activation of which in pancreatic β-cells, leads to insulin secretion (25, 47), suggesting that this receptor might be involved in the chain of events that regulate the insulin response. Importantly, Steneberg et al. demonstrated that FFAR1 overexpression impairs β-cells function, promoting hypoinsulinemia and diabetes following HFDs feeding (26). In addition, in immortalized and primary pancreatic β-cells, FFAR1 activation induces lipotoxicity (48). Altogether these studies suggest that, in pancreatic β-cells, the effects of FFAR1 activation appear to be dual, since it is involved in both homeostatic and pathologic effects. In addition, FFAR1 overexpression causes hypoinsulinemia and autophagy hyperactivation reduces insulin release in β-cells, suggesting that an additional crosstalk might be occurring between FFAR1 and autophagy, specifically in pancreatic β-cells. Considerably less information is available regarding FFAR1 function in the CNS, where its role is still elusive. Our data, supported by previously published studies (37), show that, within the hypothalamus, FFAR1 is only expressed by neurons and not by astrocytes and microglial cells. Dragano et al. also confirmed FFAR1 is expressed in hypothalamic neurons (both in neuropeptide Y (NPY) and POMC neurons) and acts to maintain whole body energy homeostasis by decreasing energy efficiency and reducing hypothalamic inflammation when FFAR1 is chemically activated by receptor specific agonists (38). Additional studies demonstrate that polyunsaturated fatty acids (PUFAs)-mediate FFAR1 activation in the hypothalamus to produce an anti-inflammatory and neuroprotective effect (37, 49–52). This apparent discrepancy in the effects of FFAR1 is most likely attributable to a ligand-dependent activation of distinct allosteric sites on the receptor by the different molecular structures (53). Furthermore, differences between SatFAs-mediated activation of the receptor in hypothalamic neurons *in vitro* vs. *in vivo* have not been investigated. In this study, we show PA-mediated FFAR1 activation in N43/5 hypothalamic neuronal cells reduces insulin sensitivity and the autophagic flux. Future research, using *in vivo* models, should evaluate if hypothalamic stimulation of this receptor may lead to the development of metabolic diseases, by affecting whole-body insulin sensitivity.

**Figure 6:**
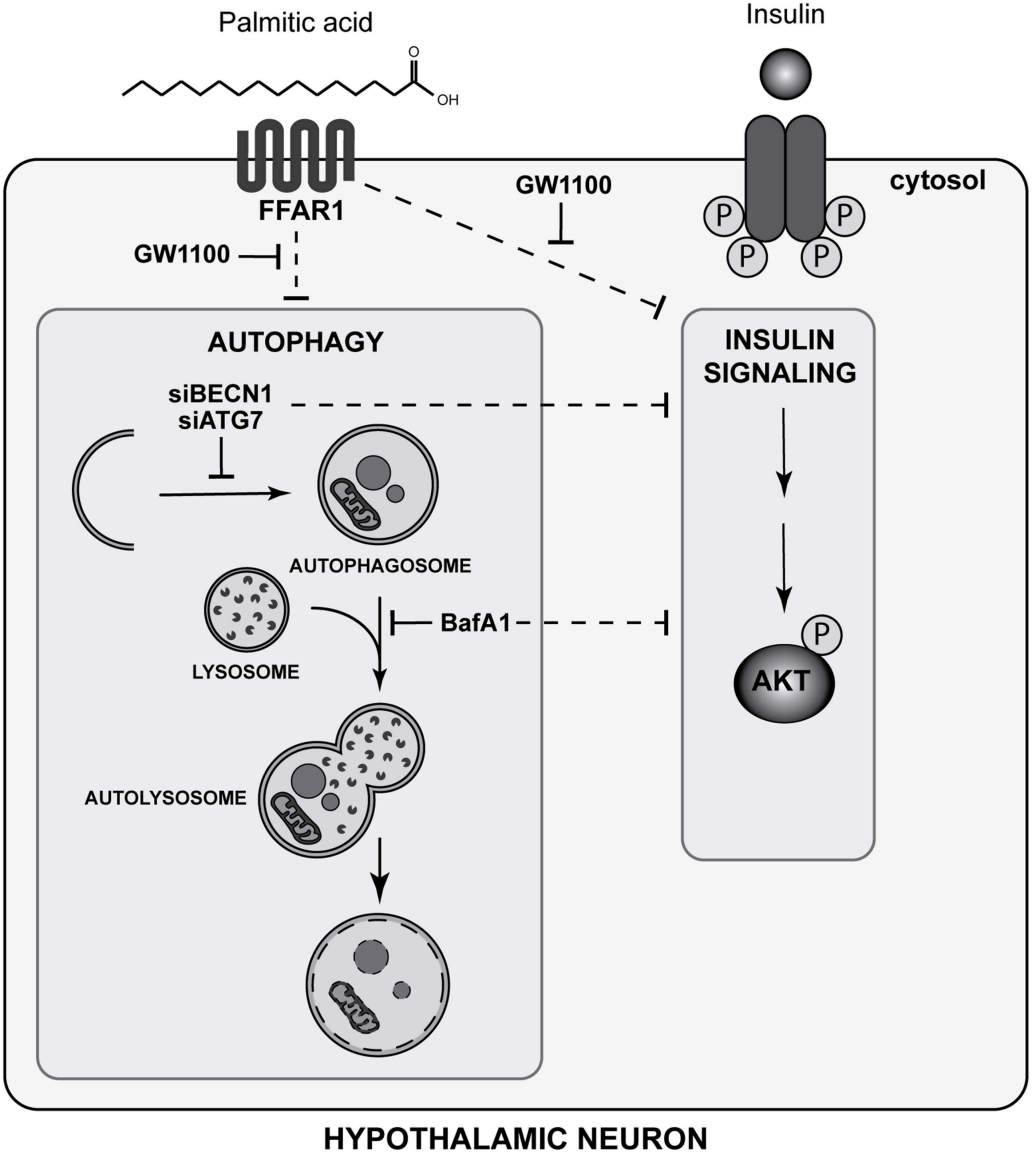
Palmitic acid-mediated FFAR1 activation decreases autophagic flux and insulin sensitivity in hypothalamic neuronal cells (Graphical abstract). The FFAR1 antagonist GW1100 prevents autophagic flux inhibition and restores insulin sensitivity, as indicated by AKT phosphorylation levels, in cells exposed to PA. Downregulation of autophagy essential genes (ATG7 and Beclin 1 -BECN1-), as well as pharmacological inhibition of the autophagic flux by BafA1, reduces the increase in AKT and IR phosphorylation levels stimulated by insulin.

In summary, our study identifies a new role of autophagy in N43/5 hypothalamic neuronal cells in the regulation of insulin sensitivity. In addition, we identified PA as negative modulator of the autophagic flux in the same cell type. Finally, our data suggest FFAR1 mediates the ability of PA to reduce insulin sensitivity promoted by autophagic flux inhibition in hypothalamic neuronal cells (Figure 6).

These results may help understanding the cellular mechanisms that drive to insulin resistance induced by PA in hypothalamic neurons.

## Author Contributions

EM and MH-C conceived and planned the experiments. MH-C, LT-V, YÁ, FD-C, CN, JE-C, and DP-O carried out the experiments. EM took the lead in writing the manuscript. All the authors contributed to the interpretation of the results and provided critical feedback and helped shape the research, analysis, and manuscript.

### Conflict of Interest Statement

The authors declare that the research was conducted in the absence of any commercial or financial relationships that could be construed as a potential conflict of interest.
