# Supplementary material for: Palmitic Acid Reduces the Autophagic Flux and Insulin Sensitivity Through the Activation of the Free Fatty Acid Receptor 1 (FFAR1) in the Hypothalamic Neuronal Cell Line N43/5
Source: Front Endocrinol (Lausanne). 2019 Mar 26;10:176. doi: 10.3389/fendo.2019.00176 (PMC6446982; doi:10.3389/fendo.2019.00176)
Supplement: Supplementary file 1 [file Data_Sheet_1.pdf]

## Supplemental Figures Legends

**Supplemental figure 1: (A)** Representative images of N43/5 cells incubated with BSA or PA for 1 h, 2 h, 4 h or 6 h and stained against LC3 (green) and SQSTM1 (red). Nuclei are stained with DAPI (blue). Bar size 10 µm. Quantification of LC3 **(B)** and SQSTM1 **(C)** dots per cell. (**D-K**) Relative mRNA gene expression of the indicated *genes* in N43/5 cells treated with BSA or PA for 1 h, 2 h, 4 h and 6 h. Data are presented as mean ± SEM. Two ways ANOVA followed by Sidak's multiple comparisons test. * $p \leq 0.05$, ** $p \leq 0.01$, ****$p< 0.0001$. n = 3.

**Supplemental figure 2**: **(A)** Representative western blot of LC3 levels in N43/5 cell lysates, incubated with vehicle (BSA), PA (100 µM) or SA (100 µM) for 6 h, in presence or absence of BafA1 (100 nM), with their respective quantifications (**B**). **(C-D)** Representative western blot of SQSTM1 levels in N43/5 cell lysates, incubated with vehicle (BSA), PA (100 µM) or SA (100 µM) for 6 h, with their respective quantifications. **(E)** Representative images of N43/5 cells treated with BSA or SA (100 µM) for 6 h in presence or absence of BafA1 (100 nM) and stained against LC3 (green) and SQSTM1 (red). Nuclei are stained with DAPI (blue). Quantification of LC3 **(F)** and SQSTM1 **(G)** dots per cell. Bar size 10 µm. Data are presented as mean ± SEM. Two ways ANOVA followed by Sidak's multiple comparisons test. *$p < 0.05$, **$p < 0.01$, ***$p < 0.001$, ****$p< 0.0001$. n= 3.

**Supplemental figure 3**: (**A**) Representative western blot showing relative levels of AKT phosphorylation (Ser473) induced by 15 min of insulin or PBS treatment in N43/5 cells pre-incubated with SA (100 µM) or BSA for 6 h, with its respective quantification **(B)**. Data are presented as mean ± SEM. Two ways ANOVA followed by Sidak's multiple comparisons test. ***$p < 0.001$, ****$p< 0.0001$. n= 3.

**Supplemental figure 4: (A)** mRNA levels of *Glut4* in N43/5 cells exposed to control (DMSO) or BafA1 for 6 h. (**B, C**) mRNA levels of *Glut 4* in N43/5 cells transfected with a siRNA to downregulate BECN1 (**B**) or ATG7 (**C**). As control condition, cells were incubated with Lipofectamine RNAiMAX reagent only (Mock). Data are presented as mean ± SEM. Unpaired t test. *$p < 0.05$. n = 3.

**Supplemental figure 5**: mRNA levels of the indicated genes in hypothalamic tissue in mice gavaged with BSA or PA for 3 days. Data are presented as mean ± SEM. Unpaired t test. . *$p < 0.05$. n = 3.

**Supplemental figure 6**: mRNA levels of *Glut4* in N43/5 cells exposed to vehicle (BSA), GW1100 or PA for 6 h as indicated. In case of GW1100 and PA treatment, cells were pre-exposed to GW1100 for 20 min, followed by GW1100 and PA exposure for 6 h. Data are presented as mean ± SEM. One way ANOVA followed by Holm Sidak’s multiple comparisons test. n = 3.

**Table 1**: Mouse primers used for real time PCR

| Gene | Forward Primer | Reverse Primer |
|---|---|---|
| *Ffar1* | TGGACAACAGTACCAGTT | GGGAGCCATTCACGGGTAT |
| *Atg7* | CTGTTCACCCAAAGTTCTTG | TCTAAGAAGGAATGTGAGGAG |
| *Sqstm1/p62* | AATGTGATCTGTGATGGTTG | GAGAGAAGCTATCAGAGAGG |
| *Lc3* | GCTCATCAAGATAATCAGACG | GCATAAACCATGTACAGGAAG |
| *Hprt1* | AAGCCTAAGATGAGCGCAAG | TTACTAGGCAGATGGCCACA |
| *Glut4* | GAGCCTGAATGCTAATGGAG | GAGAGAGAGCGTCCAATGTC |
| *Atg5* | TCAACCGGAAACTCATGGAA | CGGAACAGCTTCTGGATGAA |
| *Atg16* | AGGCGTTCGAGGAGATCATT | TTCTGCTTGTAGTTTCTGGGTCA |
| *Beclin* | TTGGGTGATGTGGGGAAAGG | AGACAGCACAGGAGGCATTC |
| *Fip200* | ACCACGCTGACATTTGACACT | CTCCATTGACCACCAGAACC |
| *Gabarap* | GCCTACAGTGATGAAAGCGTCTA | GAGCCTGAAGGAGGAACTGG |
