# Supplementary material for: Palmitic Acid Reduces the Autophagic Flux and Insulin Sensitivity Through the Activation of the Free Fatty Acid Receptor 1 (FFAR1) in the Hypothalamic Neuronal Cell Line N43/5
Source: Front Endocrinol (Lausanne). 2019 Mar 26;10:176. doi: 10.3389/fendo.2019.00176 (PMC6446982; doi:10.3389/fendo.2019.00176)
Supplement: Supplementary file 2 [file Data_Sheet_2.pdf]

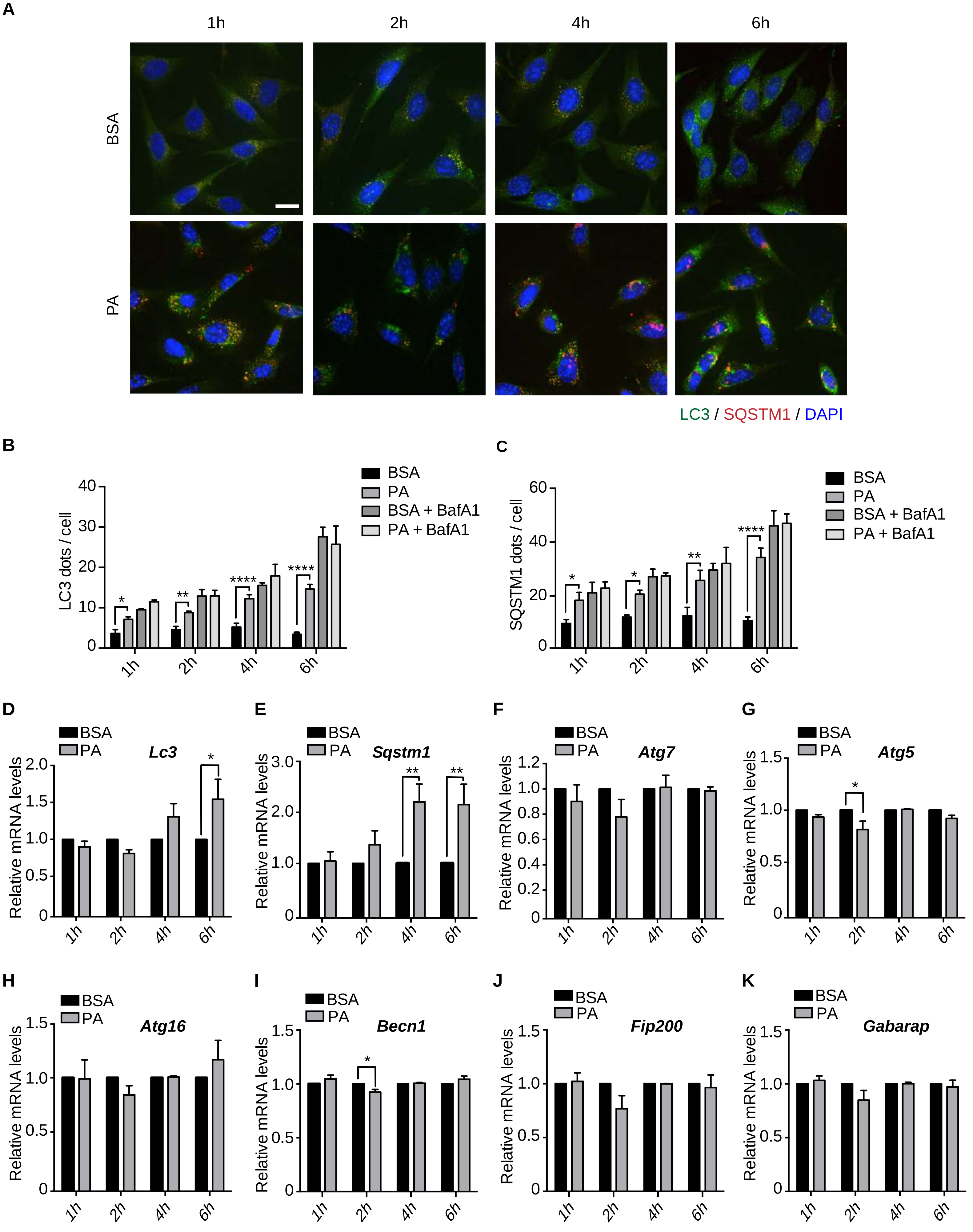

Supplemental Figure 1

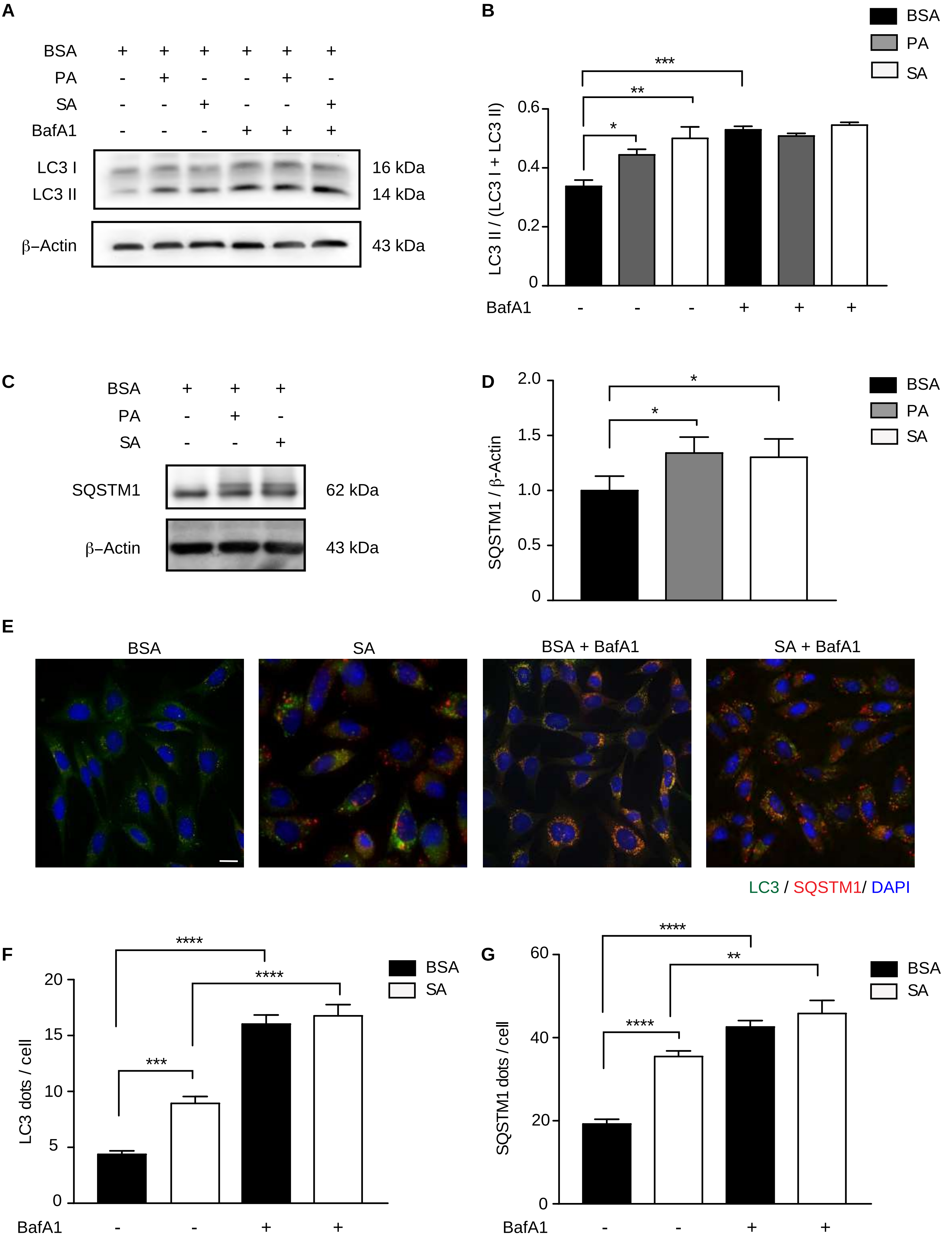

Supplemental Figure 2

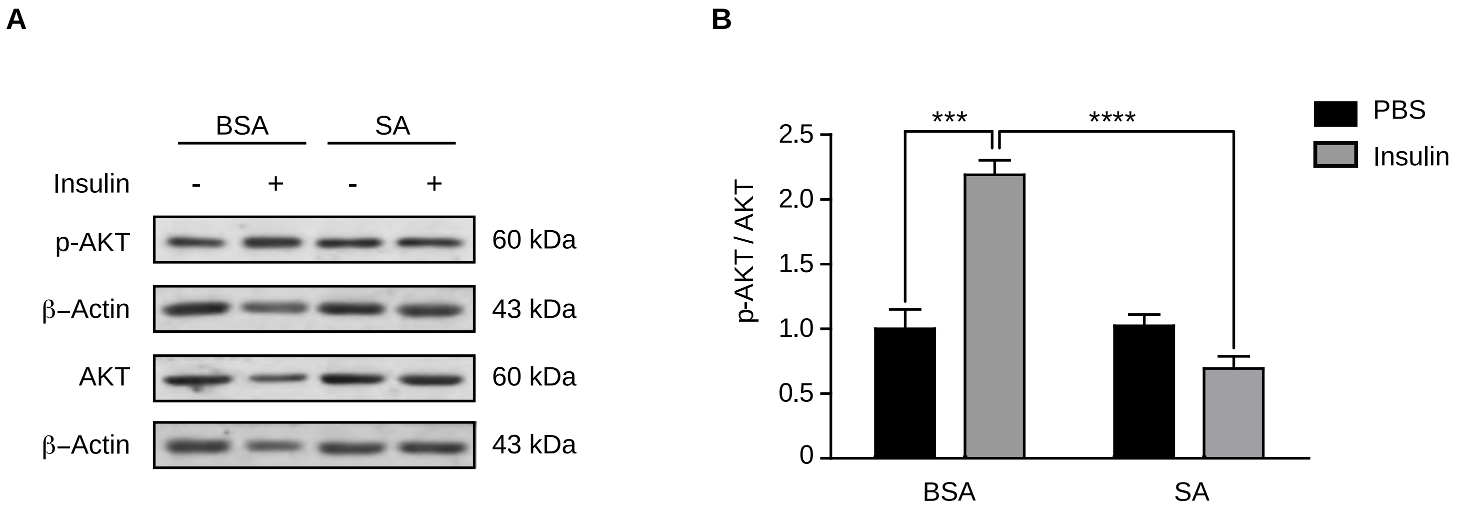

Supplemental Figure 3

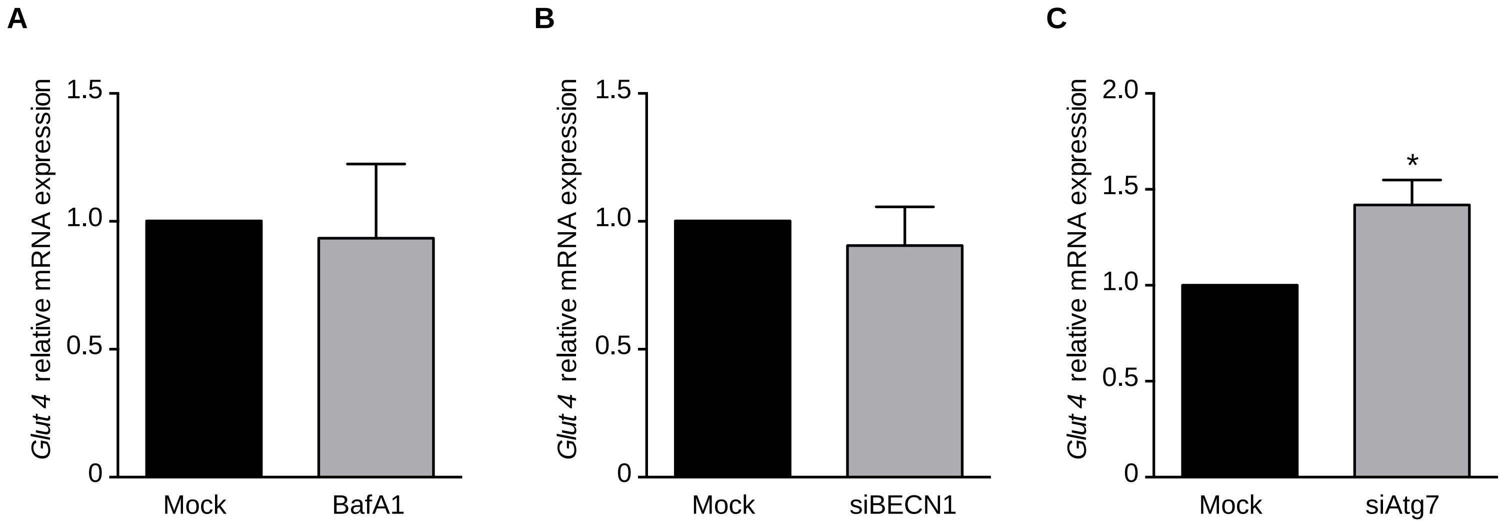

Supplemental Figure 4

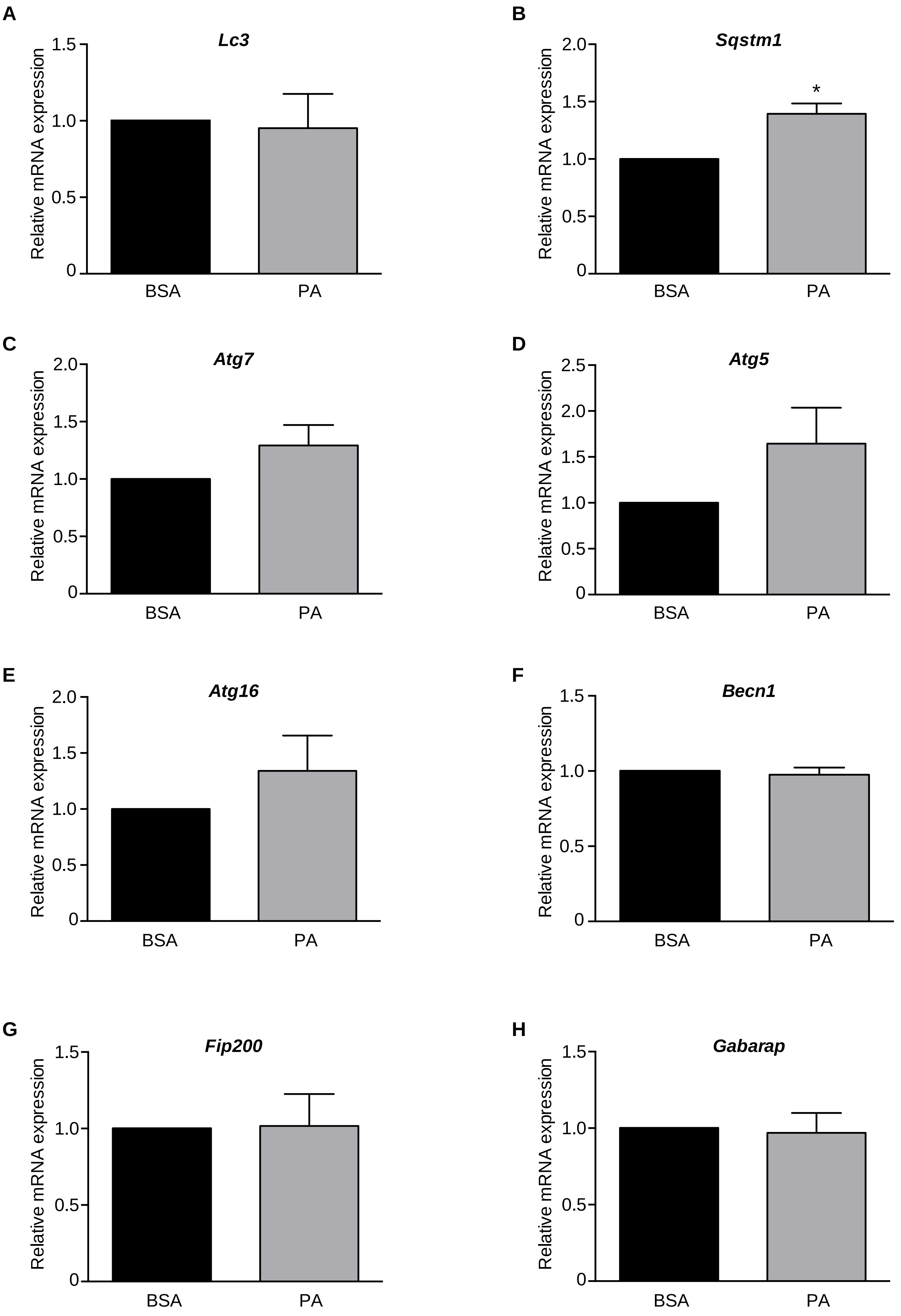

Supplemental Figure 5

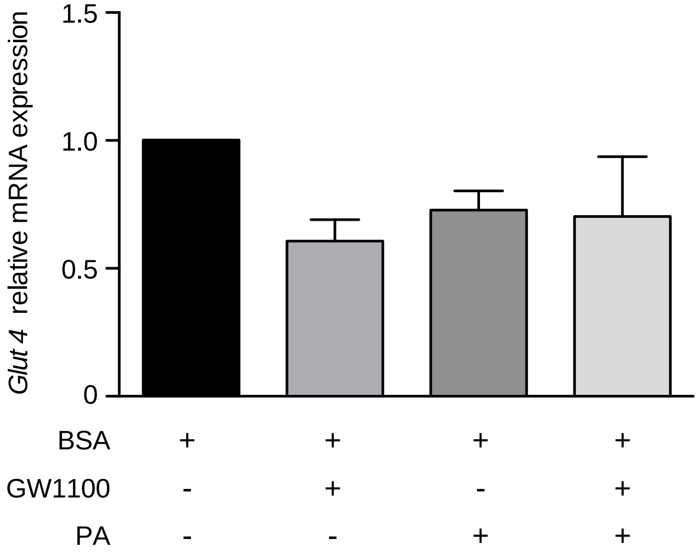

Supplemental Figure 6
